# First Report of Filamentous Phages Isolated from Tunisian Orchards to Control *Erwinia amylovora*

**DOI:** 10.3390/microorganisms8111762

**Published:** 2020-11-10

**Authors:** Ismahen Akremi, Dominique Holtappels, Wided Brabra, Mouna Jlidi, Adel Hadj Ibrahim, Manel Ben Ali, Kiandro Fortuna, Mohammed Ahmed, Bart Van Meerbeek, Ali Rhouma, Rob Lavigne, Mamdouh Ben Ali, Jeroen Wagemans

**Affiliations:** 1 Laboratory of Microbial Biotechnology, Enzymatics and Biomolecules (LBMEB), Center of Biotechnology of Sfax (CBS), University of Sfax, Road of Sidi Mansour km 6, P.O. Box 1177, Sfax 3018, Tunisia; asmahen.akremi@gmail.com (I.A.); brabra.wided@gmail.com (W.B.); jlidimanno@yahoo.fr (M.J.); adelhadjibrahim@gmail.com (A.H.I.); manel.benali@gmail.com (M.B.A.); mamdouh.benali@cbs.rnrt.tn (M.B.A.); 2 Laboratory of Gene Technology, Department of Biosystems, KU Leuven, Kasteelpark Arenberg 21-Box 2462, 3001 Leuven, Belgium; dominique.holtappels@kuleuven.be (D.H.); kiandro.fortuna@kuleuven.be (K.F.); rob.lavigne@kuleuven.be (R.L.); 3 Astrum Biotech, Business Incubator, Center of Biotechnology of Sfax (CBS), University of Sfax, Road of Sidi Mansour km 6, P.O. Box 1177, Sfax 3018, Tunisia; 4 Biomaterials Research Group (BIOMAT), Department of Oral Sciences, KU Leuven, Kapucijnenvoer 7-Block A Box 7001, 3000 Leuven, Belgium; mohammed.ahmed@kuleuven.be (M.A.); bart.vanmeerbeek@kuleuven.be (B.V.M.); 5 Department of Dental Biomaterials, Tanta University, Biomedical Campus, 32511 Tanta, Gharbia Governorate, Egypt; 6 Laboratory of Integrated Olive Production, Olive Tree Institute, BP208 Marhajene City, Tunis 1082, Tunisia; ali.rouma@gmail.com

**Keywords:** filamentous phage, Inoviridae, fire blight

## Abstract

Newly discovered *Erwinia amylovora* phages PEar1, PEar2, PEar4 and PEar6 were isolated from three different orchards in North Tunisia to study their potential as biocontrol agents. Illumina sequencing revealed that the PEar viruses carry a single-strand DNA genome between 6608 and 6801 nucleotides and belong to the Inoviridae, making them the first described filamentous phages of *E. amylovora*. Interestingly, phage-infected cells show a decreased swimming and swarming motility and a cocktail of the four phages can significantly reduce infection of *E. amylovora* in a pear bioassay, potentially making them suitable candidates for phage biocontrol.

## 1. Introduction

Fire blight, a devastating plant disease affecting a wide range of host species within the *Rosaceae* family, is caused by the Gram-negative bacterium *Erwinia amylovora*. This bacterium is one of the most important bacterial diseases affecting *Pyrus communis* (pear) and *Malus domestica* (apple) cultivars worldwide [1].

Tunisia has a long-standing tradition for the cultivation of pears in several areas across the country [2]. In the past decade, fire blight has been present in regions encompassing Mornag, Manouba and Tebourba [3]. Consequently, *E. amylovora* is causing devastating economic losses in apple and pear production in Tunisia, with a decrease from 60,000 metric tons in 2011 to less than 20,000 metric tons in 2016 for pear production alone [2]. In the past, the antibiotic streptomycin was commonly used to control *E. amylovora* during open bloom [4,5]. However, the large-scale application of antibiotics has led to the selection and increased prevalence of streptomycin-resistant strains [6]. Therefore, alternatives to control fire blight are being evaluated, such as viral biological control strategies.

Bacteriophages (phages), or bacterial viruses, are capable of targeting and killing specific bacterial hosts. Once a phage attaches to a susceptible host, it pursues a lytic or temperate replication strategy. Compared to other treatment strategies, strictly lytic phages have great potential because they are widely present in nature and they are self-replicating. Moreover, they often target bacterial receptors that are essential for pathogenesis, they are non-toxic to eukaryotes and they are specific to certain bacterial species or strains, thereby leaving beneficial bacteria unaffected. Finally, phages are relatively easy and inexpensive to isolate, produce and store [7].

Several bacteriophages of *E. amylovora* have been described and evaluated for their biocontrol potential. Gill [8] isolated 50 bacteriophages of *E. amylovora* from the Niagara region of southern Ontario in Canada. Muller et al. [9], on the other hand, characterized several *E. amylovora* phages from North America and Germany, belonging to the Podoviridae and Myoviridae families. Furthermore, in Canada, Boule et al. [10] showed that phages Ea1337-26 and Ea2345-6 reduced infection by 84% and 96%, respectively, when tested on detached pear blossoms using the epiphyte bacterium *Pantoea agglomerans* Eh21-5 as a carrier. Moreover, bacteriophage Ea2345-6, applied in combination with Eh21-5, reduced infection of fire blight on apple flowers of potted apple trees by 56%. Further, the phage carrier *P. agglomerans* extends the viability of phages on the blossom and requires the use of a lower initial phage titer. In the carrier–phage system, the critical characteristics for selecting phages as biocontrol agents are adsorption and the rate of phage production [11]. Moreover, Schwarczinger et al. [12] isolated bacteriophages which are able to infect *E. amylovora* from quince, pear and apple tissues and they applied two phages to treat apple flowers which were placed in tubes incubated in a growth chamber under 85% relative humidity. After incubation for four days, bacteria were reisolated from flowers and resulted in a bacterial decrease of at least 45%. More recently, the same group isolated phages from different sites in Hungary and they tested them in a pear slice bioassay, which yielded a 25% reduction in infection [13].

One particular family of phages, the Inoviridae or filamentous phages, are viruses capable of infecting a variety of Gram-negative and Gram-positive bacteria [14]. Filamentous phages are non-lytic nor temperate viruses that establish a persistent association with their host. During this association, the viral DNA stays present in the host cell as a double-stranded DNA plasmid, which is the replicative form (RF). Inoviridae do not lyse their host cells, but new phage particles continually exit from the bacterial host without killing it. The released virions contain a circular, positive-sense, single-stranded DNA (ssDNA), that is enclosed in a long capsid protein cylinder with a diameter of about 7 nm and a length of 800–2000 nm. The phage particles have no machinery to generate energy and parasitize their host’s existing structures in order to cross the bacterial envelope and deliver their genetic material. Typically, filamentous phages enter new hosts via pili on the cell surface [15]. The internalization of filamentous phages across the bacterial periplasmic space requires some of the components of a macrocomplex of the envelope known as the Tol system [16].

Filamentous phages are highly specific and non-lethal, but they influence their host’s fitness in several ways, which make them useful in controlling bacteria [17]. In this regard, Kawasaki et al. [18] characterized the genome of two filamentous phages, ϕRSS1 and ϕRSM1, infecting the phytopathogen *Ralstonia solanacearum*. Furthermore, Murugaiyan et al. [19] detected and isolated various kinds of filamentous bacteriophage PF226 from the rhizosphere of infected pepper, which also infect specific isolates of *R. solanacearum*. However, to our knowledge, no genome-based information has been reported for a filamentous phage that infects *Erwinia* spp.

In this manuscript, we present four filamentous phages infecting *E. amylovora*. We characterized them microbiologically and we report their genome organization. Finally, we show that a combination of them can significantly reduce infection of *E. amylovora* in a pear bioassay.

## 2. Materials and Methods

### 2.1. Bacterial Strains and Growth Conditions

The *E. amylovora* strains used in this study were isolated between 2012 and 2018 from different locations in Tunisia: Er4, Er7 and Erw from Manouba station, EaJd and Eaw from Tebourba station and Ea1 from Mornag station (Table 1). *E. amylovora* was cultured on levan agar plates (2 g/L yeast extract; 5 g/L tryptone; 5 g/L sodium chloride, 50 g/L sucrose and 15 g/L agar) or in Lysogeny Broth (LB; 5 g/L yeast extract; 10 g/L tryptone; 10 g/L sodium chloride) and, when needed, supplemented with 1% sucrose (LBS) at 25 °C.

### 2.2. Bacteriophage Isolation, Purification and Transmission Electron Microscopy

Phages were isolated from different orchards in North Tunisia: Manouba, Tebourba and Mornag. At each collection site, cuttings were taken from the aerial portions of pear trees and soil samples were taken from the bases of trees. Phage enrichment and isolation were performed according to Gill [8]. Briefly, flasks containing 60 mL of LBS (supplemented with 10 mM CaCl_2_) were inoculated with 200 µL overnight cultures of the *E. amylovora* propagation host. To each flask, 30 g of soil or 5 g of aerial tissue was added and incubated for 20 to 24 h with agitation. The mixture was centrifuged at 4 °C and 9636× *g* for 20 min. The supernatant was diluted and plated onto six bacterial lawns each, seeded with one of the propagation hosts, according to the soft agar overlay method described by Adams [20]. Lawns were checked for the formation of plaques after 24 h. Single plaques were picked from these lawns and placed into microcentrifuge tubes containing 200 µL phage buffer (10 mM Tris, 10 mM MgSO_4_ and 150 mM NaCl at pH 7.5) and 100 µL 10 mM CaCl_2_. Tubes were centrifuged at 9636× *g* and stored at 4 °C. Bacteriophage isolates were purified by passage through this single-plaque isolation procedure three times. Purified phages were stored at 4 °C.

Next, 10 µL of the purified solution was dropped on top of a 200-mesh formvar/carbon-coated copper transmission electron microscopy (TEM) grid (Structure Probe Inc., West Chester, PA, USA), left for 10 min to dry in a closed Petri dish and then contrasted by placing the TEM grid upside down on top of two drops of UranyLess (Electron Microscopy Sciences/EMS, Hatfield, PA, USA) for 10 min. After removal, the remaining solution was drained with filter paper, and the TEM grid was rinsed three times in distilled water (for 5 min) and left to dry for an additional 10 min in a closed Petri dish. Finally, the grids were additionally stained with Reynolds’ lead citrate (EMS). The bacteriophages isolates were then viewed at 80 kV on the TEM microscope (JEM 1200 EX-II, Jeol, Tokyo, Japan), and imaged with a Veleta 4k G3 camera (Emsis GmbH, Muenster, Germany), after calibration of the microscope using a 90° crossed-line calibration grid with 463-nm spacing (Grating Replica, EMS). High-resolution images were processed using Radius (Emsis GmbH).

### 2.3. Host Range Analysis

To test the host specificity, the purified phages were subjected to spot testing and plaquing assays using seven different species of the *E. amylovora* complex: *E. amylovora* Ea1, Er4, Er7, Eaw, EaJd, Erw, MG2024 and GBBC403, *Dickeya chrysanthemi* LMG 2804, *Erwinia mallotivora* LMG1271, *Pantoea agglomerans* LMG2660 and LMG 2570, *Erwinia carotovora*, *Dickeya dianthicola* LMG 2485 and *Dickeya dadantii* CFBP 3855.

In the first spot test, 5 µL of the phage suspension (10^8^ PFU/mL) was spotted on top of a double-layered levan plate for Erwinia and LB for the other bacteria. The top layer was prepared with 200 µL of test strain (optical density at 600 nm (OD_600_) of 0.8) mixed with 4 mL of LB soft agar. The plate was incubated overnight at 25 (Erwinia) or 30 °C (other genera).

All combinations showing a lysed spot were retested using a plaquing assay to rule out lysis from without. Therefore, a tenfold dilution series was prepared which was evaluated using the double agar overlay method. Here, the top layer was prepared with 200 µL of test strain (optical density at 600 nm (OD_600_) of 0.8) mixed with 4 mL of LB soft agar and 100 µL of phage dilution.

### 2.4. Temperature and pH Stability

All stability plaque assays were performed using a soft LBS agar on levan agar plates. Plaque development occurred within 24 h and assays were conducted as three biological replicates. To examine thermal stability, phage stocks (10^11^ PFU/mL in phage buffer) were incubated separately for 1, 3, 6 and 24 h at 25, 50, 80 and 100 °C. The phage titer was then evaluated using the double-layer method with early log-phase *E. amylovora* Ea1 cells. The maxima of 80 and 100 °C were tested to find out the lethal temperature for the phages, not as a relevant temperature for biocontrol application [21]. For pH stability, 10^10^ PFU/mL phage solution was added to modified phage buffer (pH values were adjusted from 3 to 11) and then incubated for 24 h at 25 °C. The resulting phage solutions were diluted to 10^4^ PFU/mL with phage buffer and were then used to infect Ea1 cells at 10^8^ CFU/mL. The plates were incubated overnight at 25 °C before plaque counting.

### 2.5. One-Step Growth Curve

A one-step growth curve was established as previously described [22], with minor modifications. The entire experiment was performed in three biological replicates. An overnight culture (4 mL) of Ea1 (OD_600_ = 0.3) was harvested by centrifugation and was resuspended in 1 mL of LBS (10^8^ CFU/mL). Phages were added to the bacterial suspension at an MOI of 0.01 and were allowed to adsorb for 10 min at 25 °C. Next, the mixture was washed with an equal volume of LBS. This manipulation was repeated three times to remove any free phage particles. After centrifugation, the pellet cells were resuspended in 1 mL of LBS and 10 µL of this culture was added to 10 mL of LBS and incubated for 2 h at 25 °C while shaking. During the incubation, samples were taken at 5 to 10 min intervals and immediately diluted, after which the dilution was plated on levan agar to determine the phage titer. The burst size was calculated as the ratio of the final phage titer to the initial number of infected bacterial cells during the latent period [20].

### 2.6. Motility Assay

Swimming and swarming motility of wildtype *E. amylovora* Ea1 was examined on LBS containing 0.3% and 0.5% (*w/v*) agar, respectively, and compared to those of PEar-infected cells. Overnight cultures of bacteria grown in LBS were centrifuged at 8000× *g* for 2 min at 4 °C, washed twice with ultrapure water and resuspended in 1 mL water (OD_600_ = 1.0). Next, 5 µL of the suspension was spotted onto LBS plates and incubated at 25 °C for five days. The migration zones were measured after 24 h for four days and used to evaluate the bacterial motility [23]. Three biological replicates were evaluated. The results are expressed as mean ± standard error of the mean (SEM). First, normality of the data was assessed using the Shapiro–Wilk test. Next, the data were statistically analyzed by one-way analysis of variance (ANOVA) to determine differences between groups using the Dunnett test. Statistical analyses were conducted using SPSS 22.0 (IBM, Armonk, NY, USA) and differences were considered statistically significant when *p* ≤ 0.001.

### 2.7. Pear Slice Assay

Effects of the phage cocktail were tested on unripe fruit slices (10 slices/treatment) of *Pyrus communis* L. conference pear cultivar, as described by Schwarczinger et al. [13]. Pears were rated after seven days for symptoms by measuring the infected area and calculating the percentage of infected tissue. Data of the pear bioassay were analyzed using JMP Pro 15 (SAS, Cary, NC, USA).

### 2.8. DNA Isolation, Manipulation and Sequencing

Phage genomic DNA (gDNA) was isolated from the phage particles using the phenol-chloroform-isoamyl alcohol method as previously described [24]. Phage DNA was digested with BglII, HhaI, BamHI, TaqI and HpoII (all Thermo Fisher Scientific, Waltham, MA, USA) for 1 h at 37 °C. Subsequently, phage DNA was analyzed with the ssDNA-specific Mung Bean nuclease (New England BioLabs, Ipswich, MA, USA) at 37 °C for 30 min. Fragments were separated on a 1% agarose gel in Tris-acetate-EDTA and stained in 1 µg/mL ethidium bromide.

Afterwards, phage RF DNA was extracted using the method of Sambrook and Russel [25]. Briefly, 1 mL of phage-infected cell culture was centrifuged before the pellet was resuspended in 100 µL of ice-cold alkaline lysis solution I (50 mM glucose, 25 mM Tris.HCl (pH 8) and 10 mM EDTA (pH 8)). After that, 200 and 150 µL of freshly prepared alkaline lysis solution II (0.2 N NaOH and 1% (*w/v*) SDS) and III (5 M potassium acetate, glacial acetic acid and H_2_O), respectively, were added and the mixture was centrifuged. Next, (1:1) phenol/chloroform was added to the supernatant of the mixture, before the RF DNA was precipitated with ethanol. The pellet was resuspended in 20 µL ultrapure water and stored at −20 °C.

The phage gDNA was sequenced on an in-house MiniSeq Illumina NGS platform (Illumina, San Diego, CA, USA). The Nextera Flex DNA library kit was used for the library prep and the average length of the DNA fragments was evaluated using an Agilent Bioanalyzer 2100 (Agilent, Santa Clara, CA, USA). The concentration was determined with Qubit (Thermo Fisher Scientific). After sequencing, Illumina reads were trimmed with Trimmomatic (v. 0.36.5) and assembled using Unicycler (v. 0.4.8.0) [26,27]. The quality of the contigs was assessed using Bandage (v. 0.8.1) and genomes were annotated using the PATRICbrc server (v. 3.6.2) [28,29]. The automated annotation was manually curated using BLASTp (National Centre for Biotechnology Information (NCBI) [30]. Alignments were made using the MUSCLE algorithm [31]. A maximum likelihood tree was constructed according to the Jones–Taylor–Thornton model with 10,000 bootstraps in UGENE [32] and MEGAX [33].

## 3. Results

### 3.1. Isolation of Six E. amylovora-Specific Filamentous Phages

**Figure 1:**
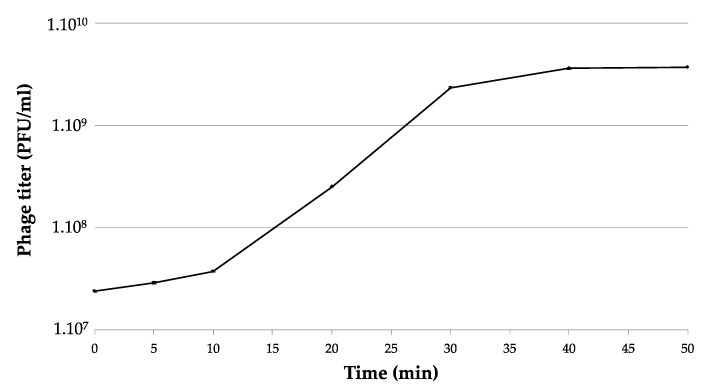
One-step growth curve of filamentous phage PEar6. The phage was added at an MOI of 0.01 and allowed to adsorb for 15 min at 25 °C. Phage titers were determined every 5 to 10 min using the plaque assay. This experiment was repeated in triplicate and standard deviations are indicated on the graph (<3.5% of the mean value).

By enrichment with Ea1, a Tunisian *E. amylovora* strain, six phages were isolated from soil and plant tissue samples collected from different orchards in North Tunisia. These were called PEar1 to PEar6 for filamentous phages of Ea1, indicated by their TEM morphology (Figure S1). Four phages (PEar1/PEar2/PEar3 and PEar5) were isolated from soil collected underneath blighted pear trees and two phages (PEar4/PEar6) from pear shoots. The isolated phages form small and turbid plaques with a diameter of 0.5–1.1 mm after incubation for 24 h at 25 °C. The six phage isolates had a similar one-step growth curve, displaying a latent period of approximately 10 min, a rise period of 25 min and an average burst size of approximately 280 phages per cell (Figure 1).

Bacterial susceptibility was characterized based on the ability of the phage to form plaques on specific hosts. Six *E. amylovora* strains, originally isolated from different regions in Tunisia, and two type strains were tested for their susceptibility to the phages. All phages could form plaques on one or more *E. amylovora* strains (Table 1). On the other hand, they could infect none of the other tested Erwiniaceae hosts, indicating their specificity.

### 3.2. Sequence Analysis Shows That PEar1-6 Resemble Filamentous Phage IKe

DNA was directly isolated from the phage particles. Unexpectedly, this DNA could not be cut by a set of diverse restriction endonuclease enzymes. However, the ssDNA Mung Bean endonuclease was able to fully degrade the DNA (Figure S2). This result indicated that the phage particles contained ssDNA, suggesting that indeed the phages in collection are indeed filamentous, consistent with other previously reported filamentous phages.

Subsequently, phage replicative-form DNA was extracted and used for genome sequencing. The genomes of the PEar phages range between 6608 and 6801 nucleotides and have a G + C content of approximately 42%. BLASTn analysis showed that these six phages are similar to the Enterobacteriaceae phage IKe (94–96% coverage, 91.96–92.06% identity), containing a 6883 nucleotides genome with a 40.55% G + C content [34]. As such, we can conclude that the PEar phages belong to the *Lineavirus* genus together with Enterobacteriaceae phages IKe and I22. PEar1 and PEar2 only differ in a few SNPs. Table 2 gives an overview of the different non-synonymous SNPs located in the genome of PEar2 compared to PEar1. The mutations are situated in gene IV and cluster together in one specific region of the protein. Other mutations are located in the non-coding regions upstream and downstream of gene IV (data not shown). PEar2, PEar3 and PEar5 appeared to be completely identical, so PEar3 and PEar5 were excluded from subsequent analyses.

**Figure 2:**
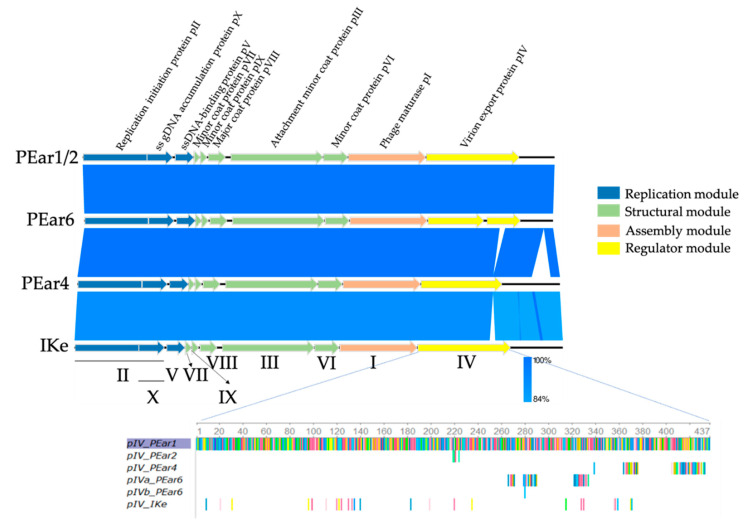
Genome maps of PEar1, PEar2, PEar4 and PEar6 and comparison between the genomes and reference phage IKe using a BLASTn analysis. The PEar phages display a modular genome organization, like that of previously described filamentous phages IKe, Fd or M13. The following four usual modules of filamentous phages are distinguishable: the replication module (blue), the structural module (green), the assembly module (orange) and the regulator module (yellow). The gene names and their encoding proteins with functional annotation (respectively at the bottom and top) follow the same numbering as their counterparts in IKe (adapted from EasyFig [37]). An alignment of the amino acid sequence (each colored bar represents a different amino acid) of pIV with PEar1 as reference shows that there are some mutations in the other sequences, indicated as colored bars. pIV from PEar4 and pIVa from PEar6 have accumulated mutations at the C-terminal end of the protein sequence.

The PEar genome maps and their homologies to IKe are presented in Figure 2. Annotation of the genome sequences of the phages revealed 10 or 11 predicted open reading frames (ORFs), all corresponding to genes described for Enterobacteriaceae virus IKe. Hence, consistent gene numbering was assigned. At the genome end, downstream of gene IV, which encodes the virion export protein pIV, an intergenic region is present, a common feature among filamentous phages [34,35].

Similar to all of the previously characterized filamentous phages, the PEar phages have a modular organization, comprised of four modules: a replication module, a structural module, an assembly module and a regulator module.

The replication module contains three ORFs: gene II, gene X which overlaps with the 3′ end of gene II and gene V. The peptide encoded by gene II/X is homologous to the pII/X protein of IKe (98.81% identity) and the replication initiation proteins of Enterobacteriaceae phages f1 and fd. Moreover, it shares identity to the corresponding protein of *Escherichia coli* virus M13 (60.44%). The protein encoded by gene V is homologous to the ssDNA-binding protein of IKe (96.59% identity) and fd (45.45%).

Within the putative structural module, we identified five ORFs: genes VII, IX, VIII, III and VI, whose encoded proteins are similar to the capsid proteins of IKe and other known filamentous phages.

In the third module of the PEar phages, the assembly module, gene I was identified, whose product is homologous to pI of IKe (97.26% identity), I22 (87% identity), If1 (56,47% identity) and M13 and Enterobacteria phage Fd (51.37% identity). These proteins are required for assembly and secretion of the viral particles [23,38].

**Figure 3:**
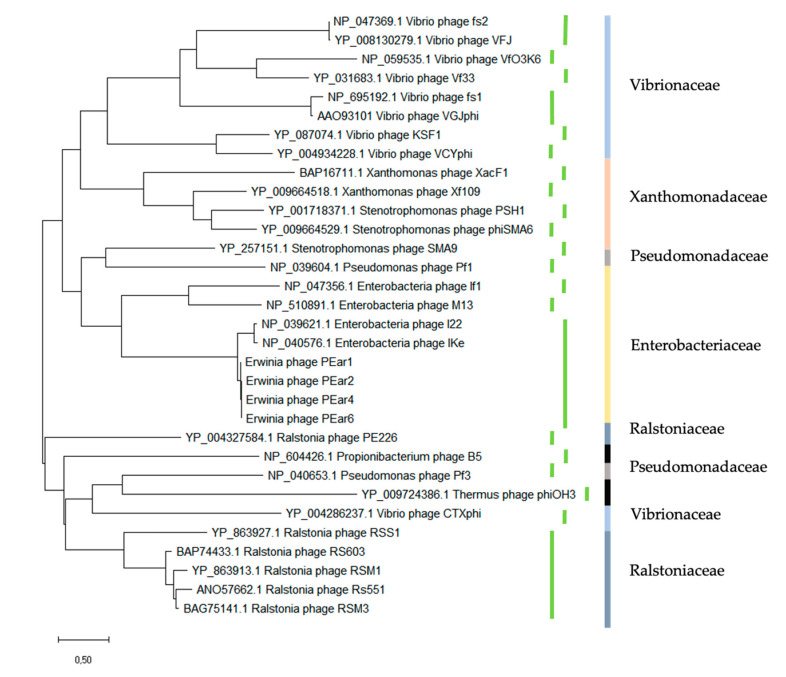
Phylogenetic analysis of filamentous phages based on the pIII amino acid sequence. Green bars represent the different genera as determined by the International Committee on Taxonomy of Viruses (ICTV). Colored bars indicate the bacterial families that the respective filamentous phage can infect (light blue Vibrionaceae, orange Xanthomonaceae, gray Pseudomonadaceae, yellow Enterbacteriaceae, dark blue Ralstoniaceae and black other bacterial families). This analysis shows that based on the pIII sequence, phages cluster according to the phylogenetic diversity of their host.

In the last part, the regulator module, we identified gene IV, which consists of two ORFs for PEar6, due to a frame shift mutation at position 686 of the gene, resulting in a premature stop codon. The second part of PEar6 gene IV is again identical to the 3′ end of the corresponding genes in the other PEar phages and IKe. An alignment of the different pIV protein sequences of the current phage collection shows that there are indeed variations among the different phages (Figure 2). These mutations are primarily located at the *C*-terminal end of the protein. In the case of PEar1 and PEar2, these mutations are located centrally within the protein. The peptides encoded by gene IV exhibit homology to several predicted transcriptional regulators of phages and the virion export protein of Ike (92.58% of identity). Intriguingly, the pIV product also displays partial similarity to Ike pIII, the minor coat protein (88% identity over 118 residues), and I22 pIII (90% identity over 106 residues). Peeters et al. [34] indeed showed that an intriguing feature of the gene III sequences of IKe is that defined stretches of coding sequences have been translocated during the evolution of these phages. A protein alignment of the pIII protein sequence of the PEar phages shows that these proteins are highly similar among these Erwinia phages (data not shown). Moreover, a phylogenetic analysis of the pIII protein of the PEar phages together with the different filamentous phages as described by the International Committee on Taxonomy of Viruses (ICTV) demonstrates that the different phages seem to cluster more or less together according to the bacterial host they parasitize (Figure 3). As such, the phylogeny of filamentous phages based on the pIII protein sequence follows the phylogeny of the bacteria according to their 16S rRNA sequence. This observation has previously been made by Mai-Prochnow et al. based on the pVIII protein sequence as well [39]. However, recently, Hay and Lithgow reported a phylogenetic study based on the pI protein sequence. Here, this clustering according to the bacterial phylogeny was less pronounced [40].

### 3.3. Temperature and pH Stability Makes the Isolated Phages Suitable as Biocontrol Agents

Supplementary Figure S3 shows the impact of temperature and pH on the infectivity of the PEar phages. They remain stable from 25 to 50 °C, since their titer at that temperature range remains constant at approximately 10^10^ PFU/mL. Interestingly, a significant loss in phage titer was only observed at 80 and 100 °C after 1 to 6 h incubation and the phages were completely inactivated at 80 and 100 °C after 24 h (Figure S3A). The crude suspension of PEar1–6, after removal of the cells, is stable for at least six months at 4 °C (data not shown).

Considering pH stability, the test shows that the PEar phages are stable between pH 3 and 9. At higher pH values, phage survival decreased substantially (Figure S3B).

### 3.4. PEar Phages Influence the Swimming and Swarming Motility of Their Host Drastically

**Figure 4:**
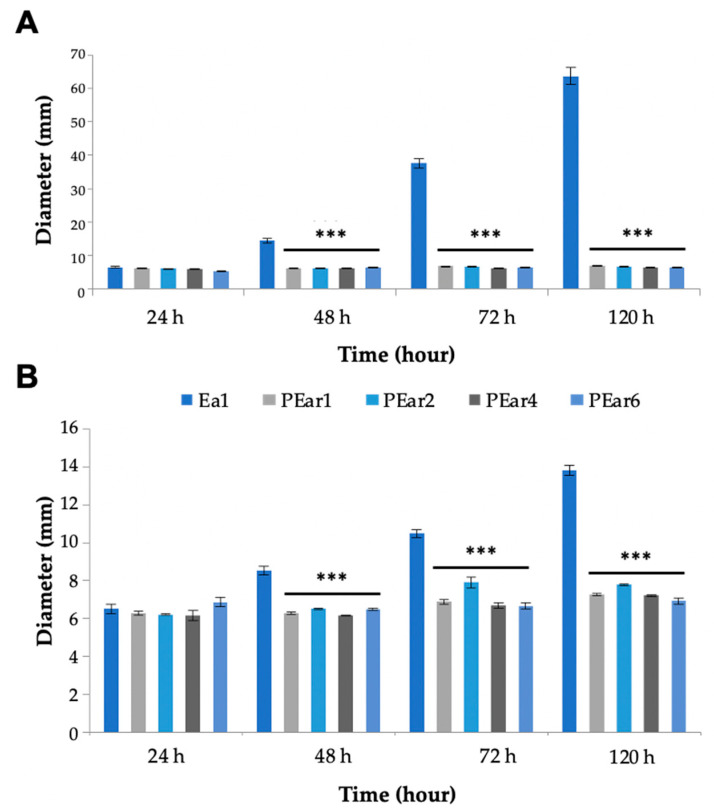
Swimming and swarming plates are affected by PEar infection. *E. amylovora* motility ((**A**)—swimming motility; (**B**)—swarming motility) was evaluated 24, 48, 72 and 120 h after infection with the different PEar phages. An uninfected strain was taken as control. Bars represent the mean of three replicates ± SEM. The asterisks denote significant differences between the treatment groups and the control group (*p* ≤ 0.001).

Swimming and swarming are two different types of motility in bacteria that require the presence of bacterial flagella. They are influenced by environmental factors such as exopolysaccharide EPS and chemotaxis [41]. Swimming and swarming motility of *E. amylovora* Ea1 was assessed in the presence or absence of phage for five days (Figure 4). This assay indicated that the swimming motility of the strain infected with the different bacteriophages was much lower in comparison with the uninfected strain (Figure 4A). Similarly, the swarming motility of the strain infected with the bacteriophages was about 50% less compared to the uninfected Ea1 (Figure 4B).

### 3.5. A Pear Slice Assay Shows the Potential of These Filamentous Phages to Control Fire Blight

Finally, a bioassay was performed to determine the efficiency of the phage cocktail by assessing fire blight symptom development in pear slices. After infection by *E. amylovora*, pear slices show maceration.

The different filamentous phages alone as monotreatment had a rather limited effect on the growth reduction of *E. amylovora* on pear slices. However, the combination of all phages reduced symptom development by 40% (10^6^ PFU/mL) compared to the positive control (10^8^ CFU/mL).

**Figure 5:**
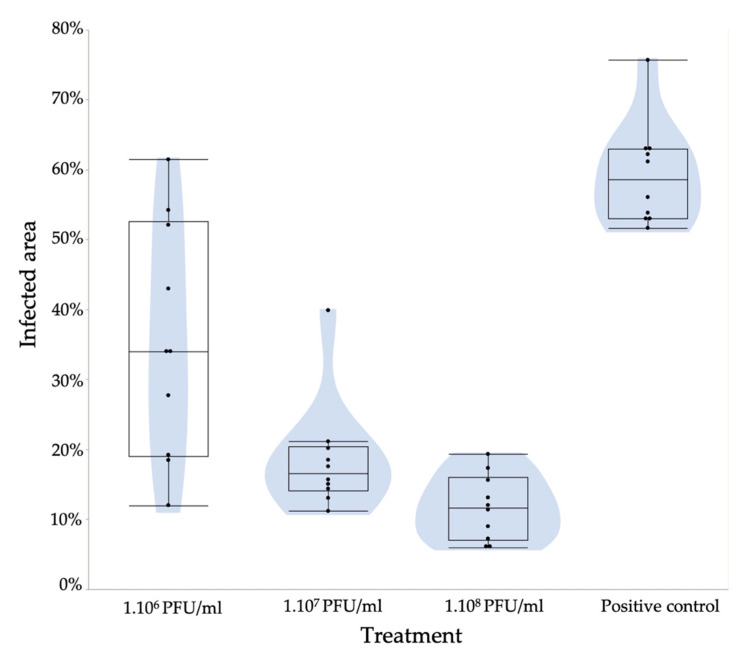
Reduction in *E. amylovora* symptoms by phage treatments on immature pear slices. Pear slices (10 slices/treatment) were treated with four phages (PEar1, PEar2, PEar4 and PEar6) with different phage concentrations (10^6^, 10^7^ and 10^8^ PFU/mL) and inoculated with 10^6^ CFU/mL of the *E. amylovora* Ea1 strain. The pear slices were soaked in the mixture of phages and inoculated with *Erwinia* after drying. This graph shows for every treatment a box/violin plot of the infection surface. At 10^7^/10^8^ and 10^6^ PFU/mL, the reduction is significant, with a *p*-value of 0.002 and 0.0036, respectively.

The results show that different concentrations (10^6^, 10^7^ and 10^8^ PFU/mL) of phage treatment of pear slices are able to increasingly reduce tissue maceration by *E. amylovora* (Figure 5). At 10^7^/10^8^ and 10^6^ PFU/mL, this reduction is significant, with a *p*-value of 0.002 and 0.0036, respectively. Our results prove that bacterial growth and motility are drastically limited in pear slices with this cocktail of four different filamentous phages, thus showing the potential of pre-treatment with filamentous phages to prevent fire blight disease.

## 4. Discussion

Given the global uncertainty around food security, there is a major effort to understand how phages can shape microbial communities to disadvantage pathogenic bacteria on crop plants. To date, several *E. amylovora* phages, all belonging to the Podoviridae and the Myoviridae families, have been isolated from different orchards of pear and apple around the world, and were shown to decrease bacterial virulence [8,10,13].

In this study, we isolated and characterized four bacteriophages (PEar1, PEar2, PEar4 and PEar6), infecting Tunisian *E. amylovora* strains. These phages appeared to be the first described filamentous *E. amylovora* phages, belonging to the *Lineavirus* genus and resembling Enterobacteria phage IKe. During multiplication of the filamentous phage, progeny virions extrude into the medium without retarding host cell growth, thereby generating turbid plaques. In terms of host range, PEar6 has a slightly broader host range compared to the other phages presented here.

### 4.1. Understanding the Genome Organization of the PEar Phages

As is the case with all Inoviridae, the PEar genomes are a single-stranded DNA molecule that is converted to a double-stranded replicative form in the host cell, which can be recovered as a plasmid. The PEar phages display a modular genome organization, like that of previously described filamentous phages IKe, Fd or M13. The following four usual modules of filamentous phages are also distinguishable in the PEar phages: the replication module, the structural module, the assembly module and the regulator module.

The pIII structural protein was previously shown to be essential for host cell recognition and virus entry, by binding the retracting pilus and the TolA receptor of the host cell, thus facilitating adsorption [42]. Consequently, phage infection is primarily mediated by this protein. It is located at one end of the phage particle and consists of three domains [43]. Upon pilus binding and retraction, the N2 domain of pIII appears to have a crucial role in assisting the infection process [40].

The gene encoding pII/X is necessary for the double-stranded DNA replication of the distantly related filamentous phages IKe and Ff (M13, fd and fl) [34]. After one replication round, pII nicks the elongated viral strand always at exactly the same position, thereby releasing the displaced parental viral strand from the newly formed dsDNA. pII has been shown to be the only phage protein product required for replication, so a plasmid containing both gene II and a strand transferase which binds to the newly synthesized supercoiled RF at the (+) strand origin is able to replicate in a host cell [34,44]. The first crucial aspect of the initial function of RF is that it serves as a template for the transcription of initial mRNA transcripts encoding phage proteins, including pII and pX, which are necessary for the amplification of the phage genome. The protein pX is identical to the C-terminal third of pII and translated from an internal start codon in the pII gene—which appears to play an additional regulatory role, although this is unclear in the relationship of F1 and RF levels [45,46].

Furthermore, pIV is an integral outer membrane protein required for both assembly and extrusion [47]. There are some observations that suggested that pIV might serve as an exit pore through which phages pass to cross the outer membrane, since after its synthesis, pIV is first secreted into the periplasm, from where it integrates into the outer membrane by forming a multimer of 12–14 subunits [48], and while the carboxy-terminal domain is incorporated into the outer membrane, the periplasmic domain of pI/pXI [49] interacts with the amino-terminal half of pIV which is exposed to the periplasm.

pV which presents the ssDNA-binding protein is used to spread a large number of viral DNA molecules prior to assembly. In the circular genome of the closely related Ff filamentous phages f1, fd and M13, genes V and VII are also positioned as an adjacent pair, bridging the replication and structural proteins modules [50]. pV plays a role in synchronizing the infection cycle in the host and in coordinating the level of F1 for conditioning. Late in infection, increasing levels of pV directly inhibit both negative strand synthesis and translation of the pII and pX proteins, resulting in accumulation of phage F1 DNA [51].

The shell of most homologous phage IKe is comprised of about 3100 copies of the IKe major coat protein [52], so we can assume this is more or less the same for PEar, since their structural modules are highly similar. During the early stages of phage infection, the major capsid protein pVIII becomes one of the most abundant proteins in the cytoplasm with more than four million copies per cell for IKe. A structural comparison of the main envelope proteins of different filamentous bacteriophages shows that the structure of Ike pVIII is very similar to that of the major capsid protein of M13, despite large sequence deviations and a difference in length, which is mainly due to the longer *N*-terminal region of Ike pVIII [52].

The assembly of filamentous phage particles is initiated by minor capsid proteins pVII and pIX which are small hydrophobic proteins, integrated into the bacterial inner membrane.

Ivey-Hoyle and Steege [53] showed that the gene VII initiation site is inactive when present on itself, but coupling it acquires approximately 10% of the translational activity from upstream gene V. The nature and distribution of mutations required for independent activity suggested that the gene VII site was intrinsically defective and throughout lacked sequences required for ribosome binding [53].

Gene I encodes pI, which is incorporated into the membrane by a signal anchoring domain, roughly leaving the N-terminal 250 residues in the bacterial cytoplasm and the *C*-terminal 80 residues in the periplasm. The cytoplasmic domain of pI is expected to act as an ATPase, fueling phage assembly and transport across the envelope [40].

### 4.2. The Use of Filamentous Phages as Biotechnological Tools

In biotechnology, the importance of viral particles has increased in recent years as they have been used in applications including molecular library screening, drug delivery, vaccine development or diagnostics [54,55].

Currently, the related *E. coli* inovirus M13 has applications in phage display technology for antibody development and bionanomaterial construction, as well as biosensors development [56,57]. Through genetic engineering, each of the phage’s proteins can be modified to display a sequence of interest, which alters the chemistry and affinity of the phage surface. A similar use of the PEar phages could be envisioned, or its genome could, e.g., be used as a cloning vector for *Erwinia* spp. and potentially other related species.

### 4.3. Assessing the Potential of PEar Phages as Biocontrol Agents

*E. amylovora* uses flagellar motility to swim from stigma tips to the hypanthium and through nectar and it has been suggested that flagella play an important role in virulence [55,58]. Our data suggest that filamentous phage-infected cells show a decreased swimming and swarming motility, which constitutes important virulence traits in *E. amylovora*. Ahmad et al. [23] demonstrated that the virulence of ϕRs551-infected *R. solanacearum* was also significantly reduced, although the reduced virulence is different from the loss of virulence caused by infection with the filamentous RSM-type phage ϕRSM3. Moreover, Addy et al. [59] described an extreme reduction in pathogenicity of the infected *Ralstonia* sp. as well as a reduction in twitching motility.

Many virulence determinants of *E. amylovora* have been characterized, including the exopolysaccharide (EPS) amylovoran, the Type III secretion system (T3SS), motility and biofilm formation. Zeng and Sundin [60] showed that in *E. amylovora*, sRNAs, among others, regulate exopolysaccharide production, surface attachment, swimming motility and virulence in apple shoot and immature pear infection models. Each of the three RNAs ArcZ, OmrAB and RmaA has been shown to regulate swimming motility behavior in *E. amylovora* [59], but the mechanisms conferring this regulatory function remain unknown. Likewise, it is not known whether the regulatory mechanisms of each sRNA are similar, such that they are functionally redundant, or whether each regulates motility in a unique way. However, in 2019, Schachterle et al. [61] showed that the same sRNAs are all required for wildtype mobility and swarming levels in *E. amylovora*. They also demonstrated that the loss of Hfq-dependent RNAs causes a change in the distribution of the population from swimming cells to slower swimming cells. Jian et al. [62] also characterized a novel promoter structure and proposed an RNA-based regulatory model for deep-sea filamentous phage SW1 production. Similarly, our filamentous phage might be affecting the regulatory mechanisms of the different sRNAs of *E. amylovora* to influence virulence.

Since our phages drastically reduced swimming and swarming in *E. amylovora*, we evaluated their potential as biocontrol agents by measuring their effect in a pear slice bioassay. The filamentous phage cocktail did indeed significantly reduce fire blight symptom development compared to a control without phage treatment. Schwarczinger et al. [13] used the same method to evaluate lytic phage biocontrol effects against *Erwinia*. They showed that a combination of three phages (ϕEaH2A, ϕEaH5K and ϕEaH7B) also effectively limits bacterial multiplication or development of fire blight symptoms in apple blossoms. Similarly, Born et al. [54] showed that phages L1, M7, S6 and Y2 offer potential for phage-based biocontrol of fire blight.

Filamentous phages could have advantages over lytic phages. So far, open field spray treatments based on phages can be limited due to stability issues, e.g., caused by sunlight or desiccation [63]. Therefore, Svircev et al. [64] have shown that the combination of the naturally occurring Erwinia phages and *P. agglomerans*, a common orchard epiphyte that is susceptible to *Erwinia* phage infection, present an effective phage-mediated biological control system. In this system, *P. agglomerans*, referred to as the carrier, limits the growth of *E. amylovora* on the blossom through competitive exclusion and by releasing phage progeny [65]. Similarly, Erwinia cells chronically infected with filamentous phage could be a valid biocontrol strategy avoiding phage stability issues. Indeed, Ahmad et al. discovered that XacF1, a filamentous phage infecting *Xanthomonas axonopodis* pv. *citri*, not only reduces swimming, swarming and twitching motility, but also reduces colonization ability and biofilm formation [23]. The advantage of filamentous phages in this perspective is that after infection, the phage remains present in the bacterial cell, continuously releasing progeny into the environment, as such reducing the virulence of the population [39]. Sharma et al. have argued that filamentous phages could be of interest in not only converting the bacterial host’s phenotype from virulent to avirulent, but also as biotechnological tools to transfer restriction endonuclease genes or toxin-producing genes *csrA* and *ompF* genes to the host cell or display specific antigens or silver nanoparticles to combat pathogenic bacteria [17].

## 5. Conclusions

In conclusion, this study presented, for the first time, the isolation and characterization of a filamentous phage against *E. amylovora*. Analogously to M13, these lineaviruses could potentially be implemented as biotechnological tools for *Erwinia* research. Moreover, the application of a phage cocktail on pear slices presents an important result that shows their potential as biocontrol agents.

## Figures and Tables

**Table 1:** Host specificity of isolated phages on various strains. The sensitivity of different related bacteria of *E. amylovora* and some different bacteria to PEar phages was evaluated by plaquing. + (colored in grey): turbid plaques; - (no color): no formation of plaques.

Species	Strain	PEar1	PEar2	PEar3	PEar4	PEar5	PEar6
***Erwinia amylovora***	Ea1	+	+	+	+	+	+
	Er4	-	-	-	+	-	+
	Er7	-	-	-	-	-	+
	Eaw	-	+	+	+	+	+
	EaJd	+	-	-	-	-	+
	Erw	-	-	-	-	-	+
	LMG 2024	-	-	-	+	-	+
	GBBC 403	+	+	+	-	+	+
***Erwinia mallotivora***	LMG 1271	-	-	-	-	-	-
***Pantoea agglomerans***	LMG 2660	-	-	-	-	-	-
	LMG 2570	-	-	-	-	-	-
***Erwinia carotovora***	sp.	-	-	-	-	-	-
***Dickeya dianthicola***	LMG 2485	-	-	-	-	-	-
***Dickeya dadantii***	CFBP 3855	-	-	-	-	-	-

**Table 2:** Overview of the non-synonymous SNPs of PEar2 compared to PEar1 as determined with iVar [36]. The frequency of the specific mutation is given as well as the e-value. Mutations are located in the gene encoding pIV and cluster together in one region inside the amino acid sequence.

Mutation (Amino Acid)	Gene Product	Frequency of Mutation (%)	e-Value
**L^219^ → F^219^**	pIV	59.6	1.81865e-217
**T^220^ → F^220^**	pIV	59.8	1.00557e-239
**N^221^ → G^221^**	pIV	66.2	0
**D^224^ → Y^224^**	pIV	85	0

## Data Availability

The PEar genome sequences were deposited in NCBI. The accession numbers are MT901797 (PEar1), MT901798 (PEar2), MT901799 (PEar4) and MT901800 (PEar6).

## Supplementary Materials

The following are available online at https://www.mdpi.com/2076-2607/8/11/1762/s1, Figure S1: TEM photomicrograph of PEar4, Figure S2: Agarose gel electrophoresis of phages PEar6, PEar4 and PEar2 genomic DNA and the digestion of ssDNA of phages with Mung Bean nuclease, Figure S3: Heat and pH stability of the PEar phages.
