# Supplementary material for: First Report of Filamentous Phages Isolated from Tunisian Orchards to Control Erwinia amylovora
Source: Microorganisms. 2020 Nov 10;8(11):1762. doi: 10.3390/microorganisms8111762 (PMC7697814; doi:10.3390/microorganisms8111762)
Supplement: Supplementary file 1 [file microorganisms-08-01762-s001.pdf]

Supplementary material

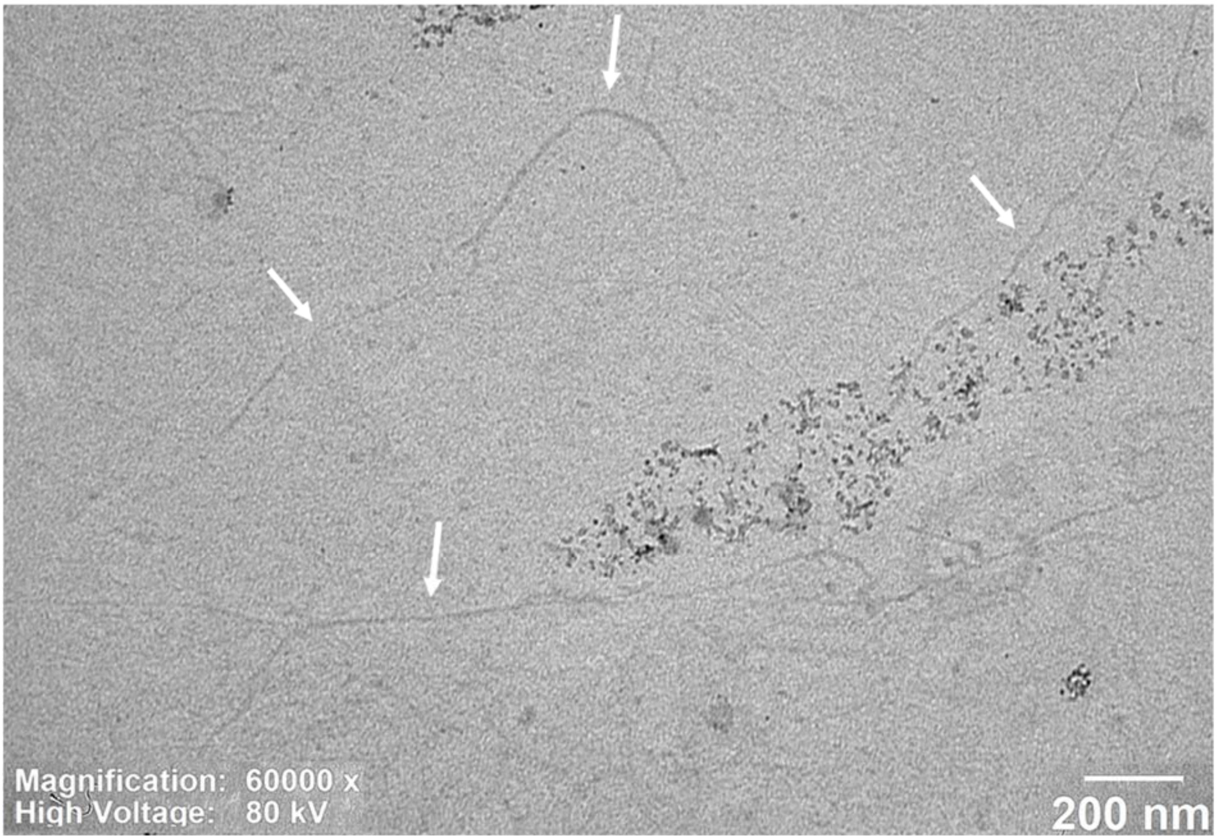

Supplementary Figure S1. TEM photomicrograph of PEar4. All PEar phages showed a similar filamentous morphology using TEM. The indicated bar represents 200 nm.

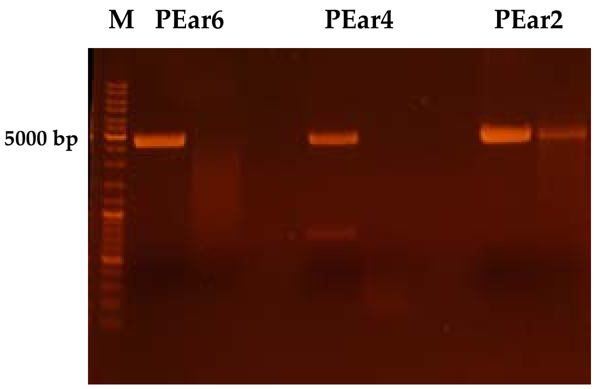

Supplementary Figure S2: Agarose gel electrophoresis of phages PEar6, PEar4 and PEar2 genomic DNA and the digestion of ssDNA of phages with Mung Bean nuclease. Lane 1 – marker, lane 2 – PEar6 DNA, lane 3 – PEar6 digested DNA, lane 4 - /, lane 5 – PEar4 DNA, lane 6 – PEar4 digested DNA, lane 7 - /, lane 8 – PEar2 DNA, lane 9 – PEar2 digested DNA.

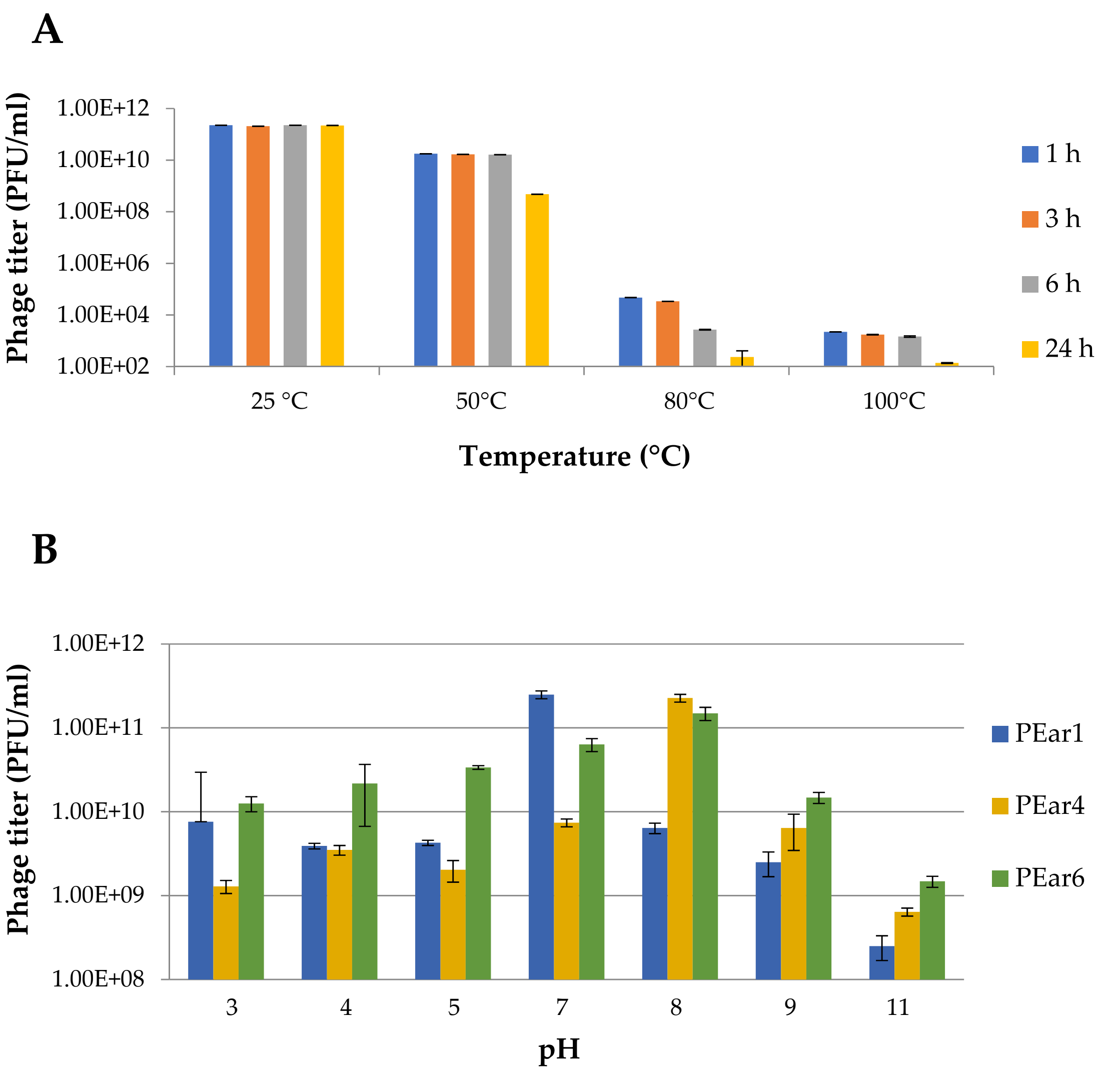

Supplementary Figure S3: Heat and pH stability of the PEar phages. (A) The PEar phages treated at different temperatures (25, 50, 80, and 100°C) with prolongation of the time from 1 hour until 24 h followed by calculating phage titer. This graph only shows the result for PEar1, since the results were similar for all the PEar phages. (B) The PEar filamentous phages treated at different pH values for one hour followed by phage titration.
